# Paracrine Effects of Adipose-Derived Stem Cells on Matrix Stiffness-Induced Cardiac Myofibroblast Differentiation via Angiotensin II Type 1 Receptor and Smad7

**DOI:** 10.1038/srep33067

**Published:** 2016-10-05

**Authors:** Kar Wey Yong, Yuhui Li, Fusheng Liu, Bin Gao, Tian Jian Lu, Wan Abu Bakar Wan Abas, Wan Kamarul Zaman Wan Safwani, Belinda Pingguan-Murphy, Yufei Ma, Feng Xu, Guoyou Huang

**Affiliations:** 1 The Key Laboratory of Biomedical Information Engineering of Ministry of Education, School of Life Science and Technology, Xi’an Jiaotong University, Xi’an 710049, P.R. China; 2 Bioinspired Engineering and Biomechanics Center (BEBC), Xi’an Jiaotong University, Xi’an 710049, P.R. China; 3 Department of Biomedical Engineering, Faculty of Engineering, University of Malaya, Kuala Lumpur 50603, Malaysia; 4 Department of Endocrinology and Metabolism, Xijing Hospital, Fourth Military Medical University, Xi’an 710032, P.R. China

## Abstract

Human mesenchymal stem cells (hMSCs) hold great promise in cardiac fibrosis therapy, due to their potential ability of inhibiting cardiac myofibroblast differentiation (a hallmark of cardiac fibrosis). However, the mechanism involved in their effects remains elusive. To explore this, it is necessary to develop an *in vitro* cardiac fibrosis model that incorporates pore size and native tissue-mimicking matrix stiffness, which may regulate cardiac myofibroblast differentiation. In the present study, collagen coated polyacrylamide hydrogel substrates were fabricated, in which the pore size was adjusted without altering the matrix stiffness. Stiffness is shown to regulate cardiac myofibroblast differentiation independently of pore size. Substrate at a stiffness of 30 kPa, which mimics the stiffness of native fibrotic cardiac tissue, was found to induce cardiac myofibroblast differentiation to create *in vitro* cardiac fibrosis model. Conditioned medium of hMSCs was applied to the model to determine its role and inhibitory mechanism on cardiac myofibroblast differentiation. It was found that hMSCs secrete hepatocyte growth factor (HGF) to inhibit cardiac myofibroblast differentiation via downregulation of angiotensin II type 1 receptor (AT_1_R) and upregulation of Smad7. These findings would aid in establishment of the therapeutic use of hMSCs in cardiac fibrosis therapy in future.

Differentiation of cardiac fibroblasts to myofibroblasts is a hallmark of cardiac fibrosis, in which cardiac myofibroblasts actively secrete collagen, resulting in excessive accumulation of extracellular matrix (ECM) and adverse remodeling of cardiac tissues that may ultimately lead to cardiac failure^1^,^2^,^3^,^4^. Cardiac myofibroblasts are likely to be derived from quiescent cardiac fibroblasts in response to biochemical cues, *e.g.*, transforming growth factor-beta 1 (TGF-β1), endothelin-1 and angiotensin II^5^. Over the past decade, it was realized that the mechanical property of ECM (*e.g.*, stiffness) also plays an important role in cardiac myofibroblast differentiation^6^. For instance, poly(ethylene glycol) diacrylate (PEGDA) hydrogel substrates with physiologically relevant stiffness (10–20 kPa) preserve inactivated cellular phenotype of cardiac fibroblasts, whereas PEGDA substrates with stiffness mimicking fibrotic cardiac tissues (40 kPa) promote cardiac myofibroblast differentiation^7^. However, recent literature suggests that cell response (*e.g.*, cell signaling and differentiation) induced by mechanical stress provided by ECM in planar or two-dimensional (2D) culture could arise from matrix protein tethering apart from substrate stiffness^8^,^9^. For instance, collagen tethering can be manipulated through modulation of the distance between two adjacent anchoring points for collagen, by changing substrate pore size or concentration of protein-substrate linker^8^,^10^, which could induce different mechanoresponsive cellular behaviors on substrates with various stiffness^8^,^11^. Therefore, it is important to develop an *in vitro* cardiac fibrosis model that can decouple the effects of ECM stiffness and pore size on cardiac myofibroblast differentiation.

To date, cardiac fibrosis cannot be completely reversed or even stopped by surgery or currently available drug therapies (*e.g.*, anti-fibrotic agents) once it has begun^12^. With advances in regenerative medicine, undifferentiated mesenchymal stem cells (MSCs) with an ability to secrete soluble factors have been found to be capable of altering the neighboring cardiac cell behaviors (*e.g.*, myofibroblast differentiation and cardiomyocyte regeneration) through paracrine effects^13^, indicating their great potential in cardiac fibrosis therapy. The existing studies showed that MSCs could suppress cardiac fibrosis by promoting secretion of matrix metalloproteinase to degrade ECM, decreasing viability of myofibroblasts and expression of alpha smooth muscle actin (α-SMA) (defining marker of myofibroblasts)^14^,^15^,^16^,^17^,^18^. However, these studies were performed on cardiac fibroblasts cultured in their non-physiological or mechanical irrelevance condition (*e.g.*, plastic cell culture plates and glass slides). To date, the paracrine effect of MSCs on cardiac myofibroblast differentiation in conditions mimicking the stiffness of *in vivo* normal and fibrotic cardiac tissues has not been explored yet. Further, the inhibition mechanism of MSCs on cardiac myofibroblast differentiation remains elusive.

In the present study, a mechanically tunable cell culture system developed by Engler’s group^10^ was used to independently investigate the effects of substrate stiffness and pore size on cardiac myofibroblast differentiation. Normal and cardiac fibrosis models were developed, based on evaluation of cardiac myofibroblast differentiation markers such as α-SMA, collagen I (Col I), collagen III (Col III) and TGF-β1, in cardiac fibroblasts cultured on collagen coated polyacrylamide (PA) hydrogels with various stiffness (4 kPa, 13 kPa and 30 kPa) and pore sizes. Following the development of such models, conditioned medium of human adipose-derived mesenchymal stem cells (hASCs) was applied to the models to determine the role and inhibitory mechanism of hASCs on cardiac myofibroblast differentiation. A schematic diagram of the methods for the development of an *in vitro* cardiac fibrosis model and treatment with conditioned medium of hASCs in the model is depicted in Fig. 1.

## Results and Discussion

### Characterization of the PA hydrogels and cardiac fibroblasts

Atomic force microscopy (AFM) and scanning electron microscopy (SEM) were performed to determine the stiffness and pore size of PA hydrogels, respectively. Through AFM analysis, we found that by varying the ratio of acrylamide monomer and *N*,*N* methylene-bis-acrylamide (crosslinker) (MBA) crosslinker, PA hydrogel substrates with different stiffness, *i.e.*, ~4 kPa, ~13 kPa and ~30 kPa, corresponding to the stiffness of immature (1–6 kPa), normal (10–20 kPa) and fibrotic (30–70 kPa) rat cardiac tissues^19^,^20^ respectively, were observed (Supplemental Fig. 1a). Moreover, SEM images show that the pore size of the PA hydrogel substrate can be adjusted without altering the stiffness. The relative pore size increases with increasing concentration of acrylamide and decreasing concentration of MBA for the 4 kPa, 13 kPa and 30 kPa hydrogel formulations (Supplemental Fig. 1b). These results indicate that the stiffness and pore size can be independently controlled for PA hydrogel substrates, which are in accordance with the findings reported by Wen, *et al*.^10^. Thus, the above substrate can be applied to decouple the effects of substrate stiffness and pore size on cardiac myofibroblast differentiation. For cell culture, Col I was selected as the matrix protein coated on PA hydrogels as it represents the majority of ECM (~75%) in the heart^21^.

Due to limited sources of primary human cardiac fibroblasts, rat cardiac fibroblasts were selected in this study. Since neonatal heart is less prone to injury which may increase cardiac myofibroblast population^22^, therefore neonatal rat instead of adult rat was used in this study. To characterize cardiac fibroblasts isolated from neonatal rat, microscopic examination, discoidin domain-containing receptor 2 (DDR2) and α-SMA immunofluorescence staining were performed. It was observed that the isolated cells display a spindle shape and DDR2 (marker of cardiac fibroblasts) expression (Supplemental Fig. 2) with no spontaneous beating, indicating the absence of cardiomyocytes in the culture. Further, there is absence of α-SMA positive cells in the culture from day 1 till day 4, suggesting the presence of only cardiac fibroblasts without myofibroblast differentiation (Supplemental Fig. 3a). However, starting from day 5, cardiac fibroblasts were spontaneously differentiated into myofibroblasts on cell culture plate, as confirmed by the presence of α-SMA positive cells (Supplemental Fig. 3a). These results are in accordance with the findings reported in literature^23^,^24^. In addition, we found that cardiac fibroblasts eventually differentiated spontaneously into myofibroblasts on day 5 disregard the passage number of the cells (Supplemental Fig. 3b). These findings suggest that the isolated cardiac fibroblasts cultured on cell culture plate should be used before day 5.

### Effects of pore size and substrate stiffness on cardiac myofibroblast differentiation

**Figure 2:**
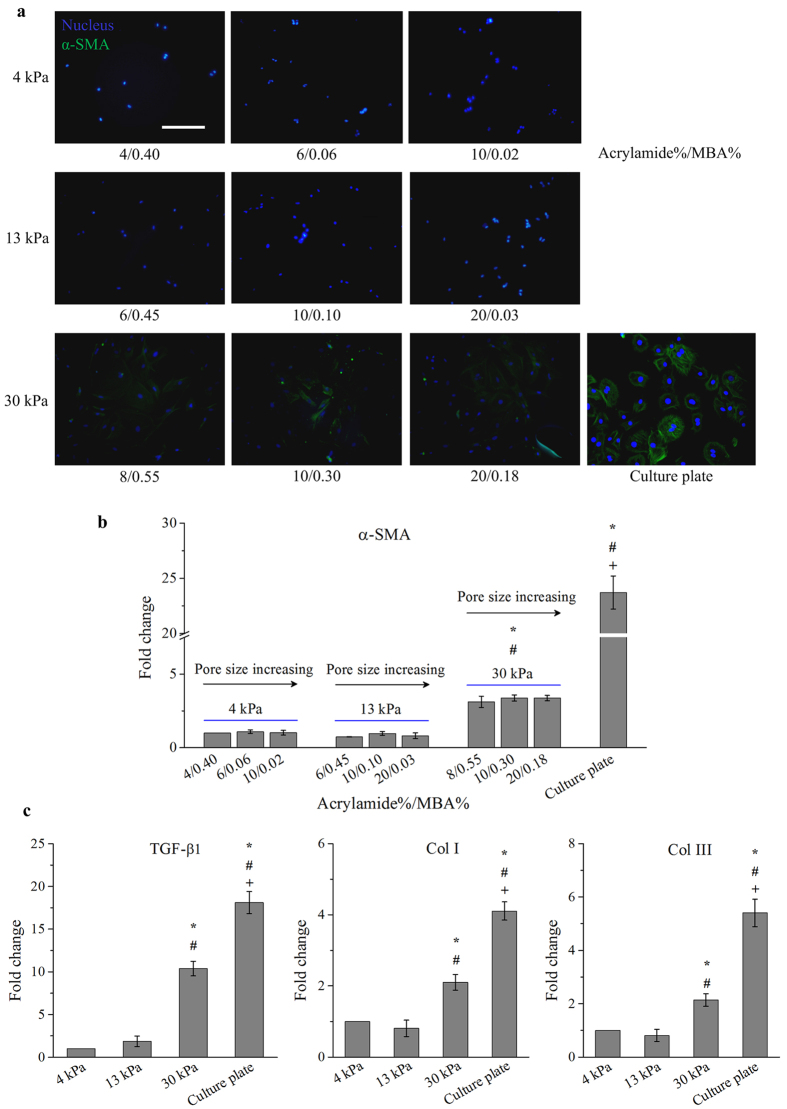
The development of an effective *in vitro* cardiac fibrosis model. Cardiac myofibroblast differentiation was dependent on substrate stiffness instead of pore size. (**a**) Cardiac fibroblasts cultured on 30 kPa substrates and cell culture plate were stained positively by α-SMA. Scale bar: 50 μm. (**b**) Varying pore sizes did not affect gene expression levels of *α-SMA* in cardiac fibroblasts. Cardiac fibroblasts cultured on 30 kPa substrates and cell culture plate display significantly (*p* < 0.05) higher gene expression levels of *α-SMA* than those cultured on 4 kPa and 13 kPa substrates. Fold change was expressed in relative to 4 kPa substrates with formulation of 4/0.4. (**c**) A significantly (*p* < 0.05) higher gene expression level of *TGF-β1*, *Col I* and *Col III* was observed in cardiac fibroblasts cultured on 30 kPa substrates and cell culture plate. Fold change was expressed in relative to 4 kPa substrates. ^*^p < 0.05 relative to 4 kPa; ^#^*p* < 0.05 relative to 13 kPa; ^+^*p* < 0.05 relative to 30 kPa. MBA: *N*,*N* methylene-bis-acrylamide.

To evaluate the effects of pore size and substrate stiffness on cardiac myofibroblast differentiation, we performed α-SMA immunofluorescence staining and gene expression analysis. We observed that there is no significant difference (*p* > 0.05) in terms of α-SMA expression among the cells cultured on PA hydrogels with various pore sizes (Fig. 2a,b). These findings suggest that varying substrate pore size or collagen tethering distance does not seem to affect cardiac myofibroblast differentiation. Similar results that indicate osteogenic and adipogenic differentiation of MSCs are not affected by such changes have been reported in literature^10^. Taken together, cardiac myofibroblast differentiation, a mechanoresponsive cellular behavior in cardiac fibroblasts, might not be regulated by pore size or collagen tethering in planar culture.

On the other hand, it was found that cardiac myofibroblast differentiation was dependent on substrate stiffness, as cells on 30 kPa substrates and cell culture plate were positive for α-SMA while those on 4 kPa and 13 kPa substrates were negative for α-SMA (Fig. 2a). Gene expression analysis shows that cardiac fibroblasts cultured on 30 kPa substrates and cell culture plate both display significantly (*p* < 0.05) higher expression levels of *α-SMA* than those cultured on 4 kPa and 13 kPa substrates (Fig. 2b), further implying that substrate stiffness plays essential role in cardiac myofibroblast differentiation. These findings suggest that cardiac myofibroblast differentiation is dependent on ECM stiffness rather than pore size.

Substrates with similar pore size but different stiffness (6/0.06 for 4 kPa, 20/0.03 for 13 kPa, and 20/0.18 for 30 kPa) were selected for further experiments. It was observed that cardiac fibroblasts cultured on 30 kPa substrates show significantly (*p* < 0.05) higher expression levels of *TGF-β1*, *Col I* and *Col III* than those cultured on 4 kPa and 13 kPa substrates, but lower than those cultured on cell culture plate (~1 GPa) (Fig. 2c). These results show that stiff ECM can induce differentiation of cardiac fibroblasts to more active cardiac myofibroblasts, which actively secrete collagen, through upregulation of TGF-β1 (a potent inducer of myofibroblast differentiation). It has been proven that myofibroblast differentiation and the secretion of collagen and TGF-β1 were elevated during cardiac fibrosis^4^,^12^. These findings suggest that cardiac fibroblasts cultured on 30 kPa substrates mimicking the stiffness of native fibrotic cardiac tissue can be used as an *in vitro* cardiac fibrosis model while those cultured on 13 kPa substrates mimicking native normal cardiac tissue can be used as an *in vitro* normal cardiac model, for pathophysiological and therapeutic studies.

### Mechanism of matrix stiffness-induced cardiac myofibroblast differentiation

**Figure 3:**
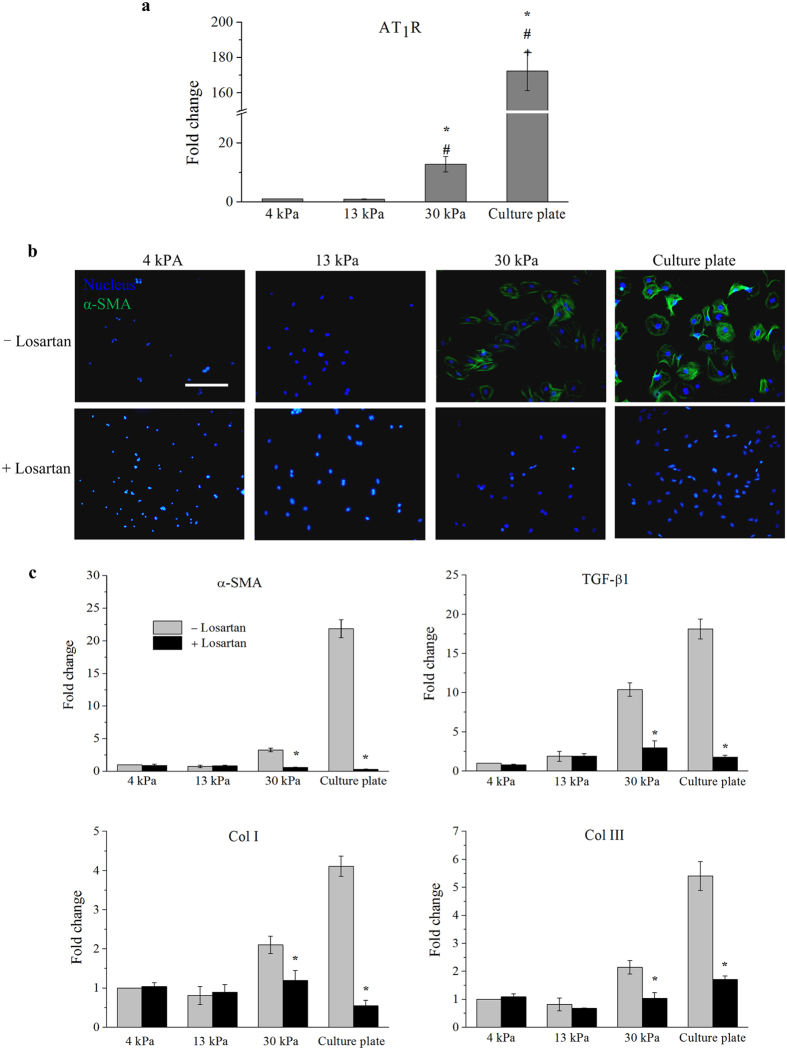
Mechanism of matrix stiffness-induced cardiac myofibroblast differentiation. Upregulation and activation of AT_1_R induced by stiff substrate enhanced cardiac myofibroblast differentiation. (**a**) A significantly (*p* < 0.05) higher gene expression level of *AT*_*1*_*R* was observed in cardiac fibroblasts cultured on 30 kPa substrates and cell culture plate (^*^p < 0.05 relative to 4 kPa; ^#^*p* < 0.05 relative to 13 kPa; ^+^*p* < 0.05 relative to 30 kPa). Fold change was expressed relative to 4 kPa substrates. (**b**) The α-SMA in cardiac fibroblasts cultured on 30 kPa and cell culture plate appeared to be reduced in the presence of losartan (AT_1_R inhibitor). Scale bar: 50 μm. (**c**) Losartan significantly (*p* < 0.05) lowered gene expression levels of *α-SMA*, *TGF-β1*, *Col I* and *Col III* in cardiac fibroblasts cultured on 30 kPa substrates and cell culture plate (*p < 0.05 relative to -losartan). Fold change was expressed in relative to 4 kPa substrates with – losartan.

To date, the comprehensive mechanism of matrix stiffness-induced cardiac myofibroblast differentiation remains elusive, and only TGF-β1 is known to enhance such differentiation in response to stiff ECM^7^,^25^,^26^. It has been proven that AT_1_R, a major receptor mediates the cardiovascular effects of angiotensin II, is activated or upregulated in cardiac fibroblasts subjected to angiotensin II to promote expression of TGF-β1 in cardiac fibroblasts to induce cardiac myofibroblast differentiation^3^,^27^,^28^. AT_1_R has also been found activated by mechanical stimuli (*e.g.*, mechanical stretch or shear stress) in the absence of angiotensin II to mediate cardiac fibrosis^29^,^30^. Cells could sense mechanical stimuli via their integrin, and thus causes active conformational change in AT_1_R to activate and upregulate AT_1_R^29^,^30^,^31^,^32^. Similarly, upregulation of AT_1_R could be seen if the cells are subjected to mechanical stress transmitted from ECM activated by its mechanical property (*e.g.*, stiffness). However, this has not been explored yet. Herein, we determined the gene expression level of *AT*_*1*_*R* in cardiac fibroblasts in response to different substrate stiffness. It was found that cardiac fibroblasts cultured on 30 kPa substrates display significantly (*p* < 0.05) higher gene expression level of *AT*_*1*_*R* than those cultured on 4 kPa and 13 kPa substrates, but lower than those cultured on cell culture plate (Fig. 3a). These results implicate that AT_1_R expression is dependent on substrate stiffness.

Further, we used losartan (AT_1_R inhibitor) to block AT_1_R-mediated signaling to determine whether AT_1_R is involved in mediating matrix stiffness-induced cardiac myofibroblast differentiation. Through immunofluorescence staining, we observed that α-SMA expression in cardiac fibroblasts cultured on 30 kPa substrates and cell culture plate is reduced in the presence of losartan (Fig. 3b). Moreover, losartan significantly (*p* < 0.05) inhibits the increase in gene expression levels of *α-SMA*, *TGF-β1*, *Col I* and *Col III* and in cardiac fibroblasts cultured on 30 kPa substrates and cell culture plate (Fig. 3c). Blocking of AT_1_R did not affect the responses of cardiac fibroblasts cultured on 4 kPa and 13 kPa substrates (Fig. 3c). These findings clearly showed that losartan acts as an inverse agonist to block the activation of AT_1_R which in turn attenuates matrix stiffness-induced cardiac myofibroblast differentiation. Losartan has been demonstrated to attenuate cardiac myofibroblast differentiation by blocking the activation of AT_1_R mediated by a mechanical stimulus (interstitial fluid shear stress)^29^.

Recently, it has been proposed that myofibroblasts may secrete angiotensin I and convert it into angiotensin II extracellularly^3^, which might work in conjunction with ECM stiffness to act on AT_1_R to induce cardiac myofibroblast differentiation. To this end, we performed enzyme-linked immunosorbent assay (ELISA) to detect the presence of angiotensin II in the conditioned medium of cardiac fibroblasts cultured on 30 kPa substrates and cell culture plate. However, we found that the concentration of angiotensin II in both the conditioned medium is 0 ng/mL, indicating the absence of angiotensin II (Supplemental Fig. 4). This indicates that AT_1_R is upregulated independent of angiotensin II. Taken together, our findings suggest that stiff ECM may induce upregulation of AT_1_R via mechanical stress-induced signaling pathway to enhance cardiac myofibroblast differentiation. In future, the mechanism by which AT_1_R expression in cardiac fibroblasts is regulated by substrate stiffness will be explored in more detail.

### Paracrine effects of hASCs on cardiac myofibroblast differentiation

**Figure 4:**
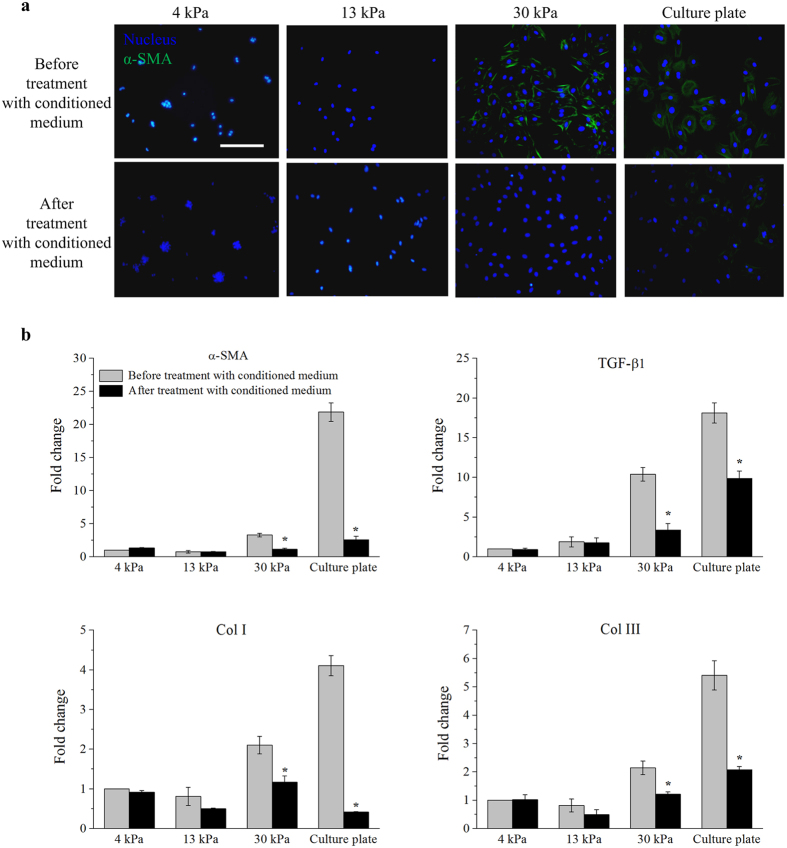
hASCs inhibited matrix stiffness-induced cardiac myofibroblast differentiation through paracrine effect. (**a**) The α-SMA in cardiac fibroblasts cultured on 30 kPa substrates and cell culture plate appeared to be reduced in the presence of conditioned medium of hASCs. Scale bar: 50 μm. (**b**) Conditioned medium of hASCs significantly (*p* < 0.05) lowered gene expression levels of *α-SMA*, *TGF-β1*, *Col I* and *Col III* in cardiac fibroblasts cultured on 30 kPa substrates and cell culture plate (*p < 0.05 relative to before treatment with conditioned medium of hASCs). Fold change was expressed in relative to 4 kPa substrates before treatment with conditioned medium of hASCs.

Among the MSCs derived from various sources of human body, hASCs have attracted special attention due to the abundance and ready accessibility of adipose tissues^33^. Analyses of the soluble factors released from hASCs have revealed that cultured hASCs at relatively early passages (within passage 5) secrete growth factors (*e.g.*, hepatocyte growth factor (HGF), Insulin growth factor (IGF)-1 and basic fibroblast growth factor (bFGF)), tumor necrosis factor (TNF)-α, cytokines (e.g., interleukin-6 and interleukin-7) and others^33^,^34^,^35^,^36^. To determine the paracrine effects of hASCs on cardiac myofibroblast differentiation, we applied conditioned medium of hASCs to rat cardiac fibroblasts for 3 days following 5-day culture on PA hydrogels. We observed that conditioned medium of hASCs reduces the expression of α-SMA in cardiac fibroblasts cultured on 30 kPa substrates and cell culture plate (Fig. 4a). Further, it was found that conditioned medium of hASCs significantly (*p* < 0.05) lowers the gene expression levels of **α-SMA*, *TGF-β1*, *Col I* and *Col III** in cardiac fibroblasts cultured on 30 kPa substrates and cell culture plate (Fig. 4b), while conditioned medium of hASCs does not have any effect on the responses of cardiac fibroblasts cultured on 4 kPa and 13 kPa substrates (Fig. 4b). It has been proven that there is a possible cross-species interaction between hASCs and rat cardiac fibroblasts, in which hASCs can reduce myofibroblast differentiation of rat cardiac fibroblasts through paracrine effects^18^. Our results indicate that conditioned medium of hASCs contains at least one kind of anti-fibrotic factor that can inhibit matrix stiffness-induced cardiac myofibroblast differentiation.

### Inhibitory mechanism of hASCs on cardiac myofibroblast differentiation

Significant effort has been put into the study of the anti-fibrotic effects of MSCs on cardiac fibrosis^14^,^15^,^16^,^17^,^18^,^37^,^38^,^39^. *In vitro* studies demonstrate that MSCs may attenuate cardiac fibrosis by degrading ECM and decreasing α-SMA expression^14^,^15^,^16^,^17^,^18^. Further, *in vivo* studies or clinical trials showed that cardiac fibrosis can be reduced when MSCs were delivered either intramyocardially or transendocardially in patients with ischemic cardiomyopathy, as indicated by improved left ventricular function and reduced scar size^38^,^39^. However, the comprehensive inhibitory mechanism of MSCs on cardiac myofibroblast differentiation remains elusive. The mechanistic investigation of anti-fibrotic effects of MSCs would help to optimize their therapeutic benefit in cardiac fibrosis therapy.

**Figure 5:**
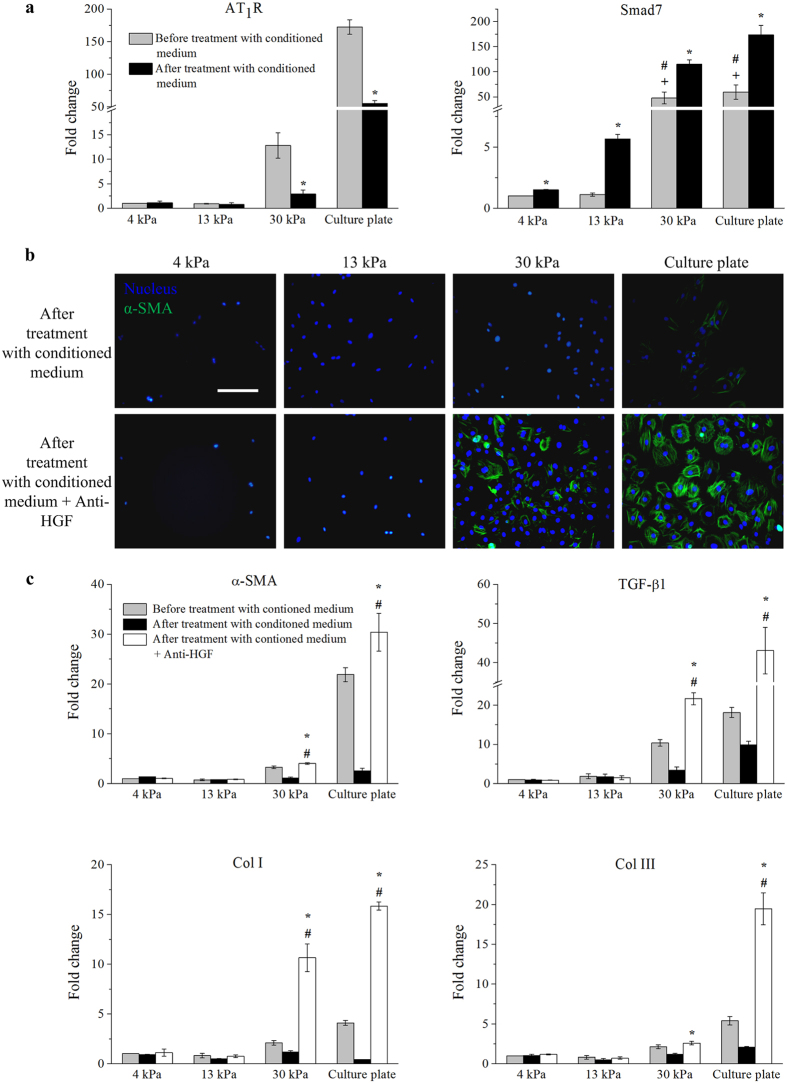
hASCs secreted HGF to inhibit cardiac myofibroblast differentiation. (**a**) Conditioned medium of hASCs significantly (*p* < 0.05) lowered the gene expression level of *AT*_*1*_*R* and enhanced the gene expression level of *Smad7* in cardiac fibroblasts cultured on 30 kPa substrates and cell culture plate (*p < 0.05 relative to before treatment with conditioned medium of hASCs; ^#^*p* < 0.05 relative to 4 kPa; ^+^*p* < 0.05 relative to 13 kPa). (**b**) HGF antibody (anti-HGF) appeared to abrogate the inhibition of conditioned medium of hASCs on the expression of α-SMA in cardiac fibroblasts cultured on 30 kPa substrates and cell culture plate. Scale bar: 50 μm. (**c**) Anti-HGF negated the anti-fibrotic activity of conditioned medium of hASCs, as indicated by significant (*p* < 0.05) higher gene expression levels of *α-SMA*, *TGF-β1*, *Col I* and *Col III* in cardiac fibroblasts cultured on 30 kPa substrates and cell culture plate (*p < 0.05 relative to after treatment with conditioned medium of hASCs; ^#^*p* < 0.05 relative to before treatment with conditioned medium of hASCs). Fold change was expressed in relative to 4 kPa substrates before treatment with conditioned medium of hASCs.

In the present study, we found that the conditioned medium of hASCs significantly (*p* < 0.05) lowers the gene expression level of *AT*_*1*_*R* in cardiac fibroblasts cultured on stiff substrates (Fig. 5a), indicating that the conditioned medium of hASCs might downregulate AT_1_R to inhibit cardiac myofibroblast differentiation. Further, it has been reported that Smad7, an inhibitory Smad, are upregulated in response to increasing TGF-β1, and blocks Smad2 phosphorylation or activation^40^,^41^ to reduce expression of α-SMA or myofibroblast differentiation^42^. We found that cardiac fibroblasts cultured on stiff substrates display a significantly (*p* < 0.05) higher gene expression level of *Smad7* than those cultured on 4 kPa and 13 kPa substrates (Fig. 5a). Moreover, with the addition of conditioned medium of hASCs, cardiac fibroblasts showed a significantly (*p* < 0.05) sharp increase in the gene expression level of *Smad7* (Fig. 5a). Taken together, these findings suggest that soluble factors in conditioned medium of hASCs might inhibit cardiac myofibroblast differentiation via downregulation of AT_1_R and upregulation of Smad7.

Several soluble factors with anti-fibrotic effects have been identified in the condition medium of MSCs, including HGF, IGF-1, bFGF and adrenomedullin^13^. HGF and IGF-1 may suppress pro-fibrotic signaling by miR-155 and miR-21 to reduce cardiac fibrosis^43^,^44^. bFGF may enhance the secretion of HGF which in turn increases the anti-fibrotic effects of MSCs^45^, whereas adrenomedullin was found to inhibit the synthesis of Col I and Col III^46^. Among the soluble factors, HGF is well-known as major contributor for anti-fibrotic function of MSCs by decreasing TGF-β1 expression and counteracting TGF-β1 signaling^47^,^48^ to reduce fibrosis in multiple organs^49^,^50^,^51^,^52^. However, the comprehensive inhibitory mechanism of soluble factors, particularly HGF, secreted by MSCs on cardiac fibrosis and myofibroblast differentiation still remains elusive. It has been reported that HGF inhibits the increase in AT_1_R expression to reduce myofibroblast differentiation of glomerular mesangial cells in the kidney^53^. Further, HGF also activates Smad7 to inhibit myofibroblast differentiation of alveolar epithelial cells in lungs^54^. Therefore, HGF could be a soluble factor in the conditioned medium of hASCs that inhibits cardiac myofibroblast differentiation via AT_1_R and Smad7. We used ELISA to determine the concentration of HGF in the conditioned medium of hASCs. A reliable standard curve of known concentration of HGF versus absorbance at 450 nm with a high coefficient of determination (R^2^ = 0.99) was plotted (Supplemental Fig. 5a). We found that concentration of HGF in conditioned medium of hASCs (135 ± 10.75 pg/mL) is significantly (*p* < 0.05) higher than that in conditioned medium of cardiac fibroblasts (0 pg/mL) (Supplemental Fig. 5b), indicating that cardiac fibroblasts did not secrete HGF. It has been reported that conditioned media collected from MSCs cultured on both tissue culture plate and substrates mimicking stiffness of native fibrotic cardiac tissues contain more HGF than those cultured on substrates mimicking physiological stiffness of cardiac tissues^55^. These findings suggest that HGF could be secreted by MSCs in response to stiff ECM, which in turn suppresses cardiac fibrosis.

**Figure 6:**
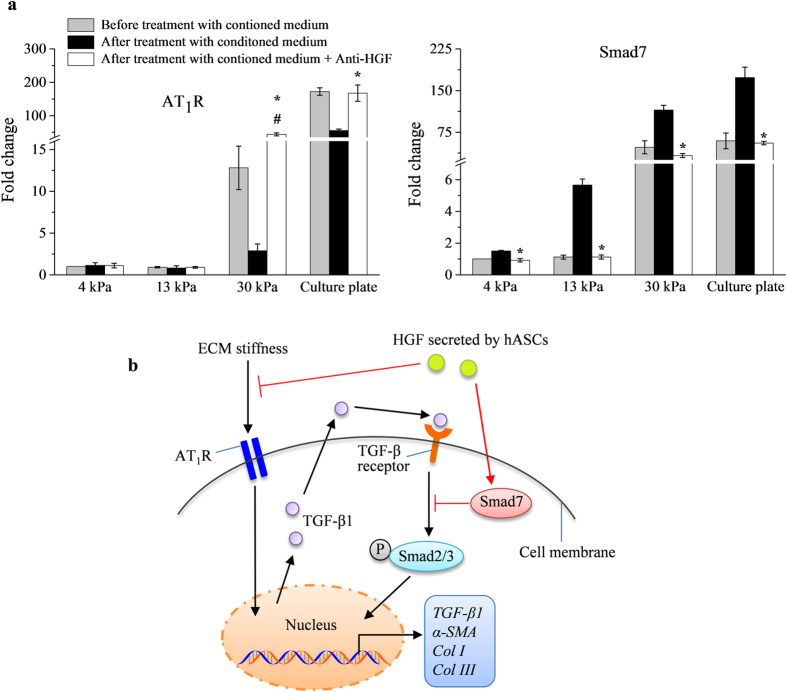
HGF secreted by hASCs abrogated cardiac myofibroblast differentiation via AT_1_R and Smad7. (**a**) Anti-HGF negated the anti-fibrotic activity of conditioned medium of hASCs, as indicated by significant (*p* < 0.05) higher gene expression levels of *AT*_*1*_*R* and lower gene expression level of *Smad7* in cardiac fibroblasts cultured on 30 kPa substrates and cell culture plate (*p < 0.05 relative to after treatment with conditioned medium of hASCs; ^#^*p* < 0.05 relative to before treatment with conditioned medium of hASCs). (**b**) Summary of proposed mechanism of matrix stiffness-induced cardiac myofibroblast differentiation and anti-fibrotic effects of undifferentiated hASCs. ECM: extracellular matrix; AT_1_R: angiotensin II type 1 receptor; HGF: hepatocyte growth factor; TGF-β1: transforming growth factor beta-1; P-Smad2: phosphorylated Smad2; α-SMA: alpha-smooth muscle actin; Col I: collagen I; Col III: collagen III.

To determine the role of HGF in the conditioned medium of hASCs for inhibition of cardiac myofibroblast differentiation, the active HGF in conditioned medium of hASCs was blocked using anti-HGF, prior to its being given to cardiac fibroblasts. Through immunofluorescence staining, we observed that cardiac fibroblasts cultured on 30 kPa substrates and cell culture plate with anti-HGF treated conditioned medium of hASCs expressed more α-SMA than those cultured with only conditioned medium of hASCs (Fig. 5b). Further, the gene expression levels of *α-SMA*, *TGF-β1*, *Col I*, *Col III* and *AT*_*1*_*R* are significantly (*p* < 0.05) higher, while the expression level of *Smad7* is significantly (*p* < 0.05) lower in cardiac fibroblasts cultured on 30 kPa substrates and cell culture plate with anti-HGF treated conditioned medium of hASCs than those cultured with only conditioned medium of hASCs (Figs 5c and 6a). These results demonstrate that HGF secreted by hASCs plays an essential role on attenuating cardiac fibrosis through downregulation of AT_1_R and upregulation of Smad7.

The proposed mechanisms for matrix stiffness-induced cardiac myofibroblast differentiation and anti-fibrosis effects of HGF secreted by hASCs are summarized in Fig. 6b. HGF-induced downregulation of AT_1_R or overexpression of Smad7 has been showed to attenuate fibrosis in multiple organs, such as kidney, lungs and gingiva, as indicated by decreased expression of TGF-β1, α-SMA and Col I^42^,^53^,^54^. It has been reported that HGF might bind to HGF receptor (c-met) to reduce AT_1_R expression via phosphatidylinositol 3-kinase/Akt (PI3K-Akt) signalling pathway. HGF activates phosphatase and tensin homolog (PTEN), a negative regulator of PI3K-Akt pathway, to inhibit the phosphorylation of Akt and thus decreases the expression of AT_1_R^53^,^56^,^57^. Meanwhile, mitogen-activated protein kinase-extracellular-signal-regulated kinase (MEK-ERK) signalling might contribute to HGF-induced upregulation of Smad7^54^. Smad7 is actively transported from the nucleus to the cytoplasm to prevent interaction of Smad2 with TGF-β1 receptor, leading to inactivation of Smad2/3 and reduced myofibroblast differentiation^41^. Further studies are needed to fully elucidate the signaling pathways on regulation of AT_1_R and Smad7 in cardiac myofibroblasts by HGF-secreted hMSCs.

It has been suggested that MSCs may counteract cardiac fibrosis through either paracrine effects, direct cell-to-cell contact or differentiation to cardiomyocyte^13^. The therapeutic effect based on cardiomyogenic differentiation potential of MSCs on cardiac fibrosis has been shown greatly reduced under stiff ECM. For instance, when undifferentiated MSCs were implanted into the heart of rat model of cardiac fibrosis post myocardial infarction, they showed bone tissue formation instead of cardiomyogenic differentiation^58^. MSCs were shown to present low expressions of early cardiac transcription factors (*e.g.*, Nkx2.5 and GATA4) in response to stiff ECM upon cardiomyogenic induction^55^. Recently, it has been showed that hMSCs exert anti-fibrotic effects by directly communicating with myofibroblasts^18^. Taken together, hMSCs may hold great promise for treating cardiac fibrosis through both paracrine effects and direct cell-to-cell contact.

## Conclusion

We demonstrate that cardiac myofibroblast differentiation is dependent on substrate stiffness instead of substrate pore size. Stiff substrate promotes the expression of AT_1_R, which in turn upregulates and activates TGF-β1 to induce cardiac myofibroblast differentiation. hASCs can secrete HGF to reduce substrate stiffness-induced cardiac myofibroblast differentiation through downregulation of AT_1_R and upregulation of Smad7. With the development of stem cell delivery method, hASCs may hold great potential for cardiac fibrosis therapy and heart regeneration in future.

## Methods

### Isolation and culture of cardiac fibroblasts and hASCs

Cardiac fibroblasts were isolated from the hearts of neonatal Sprague-Dawley rats (1–3 day old), which conform the NIH guidelines (Guide for the care and use of laboratory animals). First, heart tissues of the rats were excised following euthanasia by cervical dislocation. The heart tissues were washed with phosphate buffered saline (PBS) (MP Biomedicals, Aurora, Ohio) and minced into small pieces. Tissue digestion was performed using 0.8% collagenase type II enzyme (MP Biomedicals, Aurora, Ohio) solution at 37 °C with agitation. The digested heart tissues were centrifuged and washed to obtain the pellets. The pellets were resuspended with cell culture medium composed of Dulbecco’s Modified Eagle’s medium (DMEM)/Ham F-12 (Corning Cellgro, Manassas, USA), 10% fetal bovine serum (FBS) (Thermo Scientific, Rockford, IL) and 1% Penicillin/Streptomycin (Gibco, New York, USA), and plated for 45 mins in a cell culture plate at 37 °C and 5% CO_2_. This allows preferential attachment of cardiac fibroblasts to the cell culture plate. Then, cell culture medium containing cells (mainly cardiomyocytes) were removed and replaced with fresh cell culture medium. The isolated cells were characterized using DDR2 (marker of cardiac fibroblasts) immunofluorescence staining the next day. Cardiac fibroblasts cultured on day 1 to day 9 and passage 1 to passage 4 after isolation were subjected to α-SMA immunofluorescence staining for evaluation of cardiac myofibroblast differentiation. Based on the results, cardiac fibroblasts on day 4 after isolation were selected, and seeded on PA substrates for further experiments.

Human adipose tissues were obtained from female donors (25–35 years old) undergoing caesarean section with prior written informed consent. Isolation of hASCs was performed conform the declaration of Helsinki using protocols as described elsewhere^59^,^60^,^61^,^62^. In brief, human adipose tissues were washed, minced and digested using 0.3% collagenase type I (MP Biomedicals, Aurora, Ohio) solution at 37 °C with agitation. Cell pellets obtained after washing and centrifugation were cultured in cell culture plate with DMEM/Ham F-12 medium containing 10% FBS and 1% Penicillin/Streptomycin until passage 3. Characterization of hASCs at passage 3 has been performed in previous study^59^. Passage 3 hASCs were seeded with a cell number of 3 × 10^5^ cells/cm^2^ into a cell culture plate. Then 3 days after seeding, medium was collected and used as a conditioned medium of hASCs for evaluating the paracrine effects of hASCs on cardiac myofibroblast differentiation.

All experimental protocols involving animal and human subjects were approved by Xi’an Jiaotong University and University of Malaya ethic review board (reference no. 996.46). The methods were carried out in accordance with the approved guidelines.

### Fabrication of PA hydrogels

Glass coverslips and slides were cleaned of organics with a detergent (30 min), 100% acetone (30 min), 100% methanol (30 min) and 0.05 N NaCl (1 hour) successively, then rinsed with water and dried in an oven. The surface of a clean coverslip was functionalized with 2% 3-(trimethoxysilyl)propyl methacrylate (Sigma Aldrich, St. Louis, USA) in ethanol to enable covalent attachment of PA hydrogel substrate to glass. Meanwhile, the surface of a glass slide was treated with dichloromethylsilane (DCMS) (Sigma Aldrich, St. Louis, USA) to enhance the hydrophobic property of glass for facilitating easy detachment of PA hydrogel after polymerizing on the above treated coverslip. Fabrication of PA hydrogels was performed following the protocols as described elsewhere^10^. In brief, a polymer solution containing acrylamide (monomer) (MP Biomedicals, Aurora, Ohio), MBA (crosslinker) (Sigma Aldrich, St. Louis, USA), 1/100 volume of 10% aluminium persulfate (APS) (Sigma Aldrich, St. Louis, USA) and 1/1000 volume of *N*,*N*,*N′*,*N′*-tetramethylethylenediamine (TEMED) (Sigma Aldrich, St. Louis, USA) was prepared. This solution was sandwiched between a DCMS-treated slide and a functionalized coverslip, and allowed to polymerize at room temperature for 5 min. Following polymerization, PA hydrogel was incubated in 1 mg/mL *N*-sulphosuccinimidyl-6-(4′-azido-2′-nitrophenylamino) hexanoate (sulfo-SANPAH) (Pierce, Rockford, IL) activated with ultraviolet (UV) light for 10 min, washed with 50 mM 4-(2-hydroxyethyl)-1-piperazineethanesulfonic acid (HEPES) buffer at pH 8.5 (Sigma Aldrich, St. Louis, USA), and then incubated in 50 μg/mL rat tail collagen type I (Corning, Manassas, USA) in deionized water overnight at room temperature. Sulfo-SANPAH acts as a protein-substrate linker to couple collagen type I to the surface of PA hydrogel to facilitate cell adhesion. Collagen-coated PA substrate was kept in PBS at 4 °C and UV sterilized prior to be used for cell culture. The ratio of acrylamide (%)/MBA (%) was varied in order to adjust PA hydrogel substrate stiffness and pore size.

### PA hydrogel characterization: AFM and SEM

An AFM (Innova, Veeco, Santa Barbara, USA) was used to determine the stiffness of PA hydrogel by indentation. PA hydrogels on glass coverslip were swollen to equilibrium in PBS and indented at a velocity of 2 μm/s until a trigger of 2 nN was detected. All AFM data were analysed using SPIP 6.3.3 software (Image Metrology, Denmark) to determine the Young’s modulus, which represents the stiffness of PA hydrogel.

On the other hand, the PA hydrogels polymerized on glass coverslip were left to swell in deionized water overnight, followed by freezing in liquid nitrogen. Then, the frozen hydrogel was lyophilized overnight using a freeze dryer (Heto PowerDry LL 1500, Thermo Scientific, Rockford, IL). Lyophilized samples were sputter coated with Iridium followed by observation using a SEM (S-3000N, Hitachi, Japan). The images were taken at 700× at 15 kV.

### Evaluation of cardiac myofibroblast differentiation in cardiac fibroblasts cultured on collagen coated PA hydrogels

To determine the effect of substrate stiffness and pore size on cardiac myofibroblast differentiation, cardiac fibroblasts were seeded at a concentration of 2 × 10^5^ cells/cm^2^ on collagen-coated PA hydrogels with different ratios of acrylamide (%)/MBA (%). Cardiac fibroblasts cultured on a cell culture plate (with a stiffness of ~1 GPa) were used as a positive control. Following the 5-day culture on PA hydrogels, these cells were subjected to evaluation of cardiac myofibroblast differentiation through α-SMA immunofluorescence staining and gene (*α-SMA*, *TGF-β1*, *Col I*, *Col III*, *AT_1_R* and *Smad7*) expression analysis (described later). Further, the concentration of angiotensin II in conditioned medium of cardiac fibroblasts cultured on 30 kPa substrates and cell culture plate was determined by ELISA (described later).

In addition, to explore the role of AT_1_R in cardiac myofibroblast differentiation, losartan (AT_1_R inhibitor) (Selleckchem, Houston, USA) at 10^−7^ M was given to cardiac fibroblasts cultured on PA hydrogels on day 4. The treatment was lasted for 1 day. The cells were then subjected to cardiac myofibroblast differentiation assessment.

### Evaluation of paracrine effects of hASCs on cardiac myofibroblast differentiation

Conditioned medium of hASCs was given to cardiac fibroblasts following 5-day culture on PA hydrogels, to determine the paracrine effects of hASCs on cardiac myofibroblast differentiation. The treatment lasted for 3 days. To explore the potential role of HGF on anti-fibrotic activity of conditioned medium of hASCs, the conditioned medium was incubated with 3 ng/mL neutralizing antibody for HGF (anti-HGF) (R & D System, Minneapolis, MN, USA) at 37 °C for 1 hour prior to being given to cardiac fibroblasts. After 3 day-treatment, myofibroblast differentiation evaluation of these cells was performed. The concentration of HGF in conditioned medium of hASCs was determined by ELISA (described later).

### Immunofluorescence staining

Cells were subjected to fixation using 10% formaldehyde followed by cell membrane permeabilization with 0.5% triton X-100. Immunofluorescence staining was performed using DDR2 primary antibody (1:50) (rabbit polyclonal, Santa Cruz Biotechnology, CA, USA) and donkey anti-rabbit-TRITC secondary antibody (1:1000) (Abcam, MA, USA); α-SMA-FITC antibody (1:100) (Sigma Aldrich, St. Louis, USA); 4′-6-diamidino-2-phenylindole (DAPI) (Southern Biotechnology Associates, Birmingham, Alabama). The images were captured by a digital camera connected to an inverted fluorescence microscope (Olympus IX81, Tokyo, Japan). Image overlay was performed using ImagePro Plus 6.0 (Media cybernetics. Inc., Bethesda, MD).

### RNA extraction, cDNA sysnthesis and Real-Time PCR

Total RNA extraction was performed on cardiac fibroblasts using a total RNA extraction kit (Tiangen Biotech, Beijing, China) following the manufacturer’s instruction. RNA was converted to cDNA using a RevertAid first strand cDNA synthesis kit (Thermo Scientific, Rockford, IL) and a thermal cycler (Veriti, Applied Biosystem, Foster City, USA), with a protocol and thermal profile recommended by manufacturer. A solution containing cDNA, TaqMan gene expression assay (Applied Biosystem, Foster City, USA) and fast advanced Master Mix (Applied Biosystem, Foster City, USA) was prepared, and Real-Time PCR assay was then conducted using a Real-Time PCR system (Fast 7500, Applied Biosystem, Foster City, USA). The genes to be analyzed include *Col I* (Rn00584426_m1), *Col III* (Rn01437681_m1), *α-SMA* (Rn01759928_g1), *TGF-β1* (Rn00572010_m1), *AT*_*1*_*R* (Rn00578456_m1) and *Smad7* (Rn00578319_m1). Housekeeping gene used for normalization was *GAPDH* (Rn99999916_s1). All data were expressed as fold change in gene expression relative to a group as stated in figure legends.

### ELISA

ELISA was performed to determine the concentration of HGF in conditioned medium of hASCs and angiotensin II in conditioned medium of cardiac fibroblasts by using a human HGF ELISA kit (Neobioscience, Beijing, China) and angiotensin II ELISA kit (Phoenix Pharmaceuticals, Burlingame, CA, USA), respectively, following the manufacturer’s instruction. Absorbance of standards and samples at 450 nm were measured using a microplate reader (Multiskan G0, Thermo Scientific, Rockford, IL). Both standard curves with known concentration of HGF and angiotensin II versus absorbance at 450 nm were plotted. By referring to the standard curves, both the concentration of HGF in conditioned medium of hASCs and angiotensin II in conditioned medium of cardiac fibroblasts, which corresponds to its absorbance at 450 nm, was determined. Absorbance at 450 nm of medium without cell (free of angiotensin II and HGF) was used for normalization.

### Statistical analysis

All statistical analysis were performed with One-Way ANOVA with tukey post hoc test, independent *t*-test, or paired *t*-test accordingly using SPSS 18.0 software. Each datum was expressed as mean ± standard error of mean (s.e.m.) of at least three independent experiments (n ≥ 3). *p* < 0.05 is accepted as statistically significance.

## Additional Information

**How to cite this article**: Yong, K. W. *et al*. Paracrine Effects of Adipose-Derived Stem Cells on Matrix Stiffness-Induced Cardiac Myofibroblast Differentiation via Angiotensin II Type 1 Receptor and Smad7. *Sci. Rep.* **6**, 33067; doi: 10.1038/srep33067 (2016).

## Supplementary Material

Supplementary Information

## Figures and Tables

**Figure 1:**
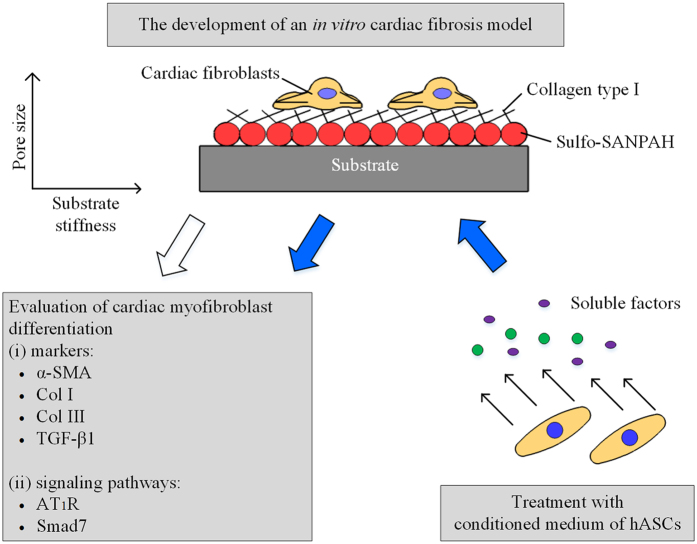
Schematic diagram demonstrates the methods for the development of an *in vitro* cardiac fibrosis model and treatment with conditioned medium of hASCs to the model. Sulfo-SANPAH: *N*-sulphosuccinimidyl-6-(4′-azido-2′-nitrophenylamino) hexanoate TGF-β1: transforming growth factor-beta 1; AT_1_R: angiotensin II type 1 receptor; hASCs: human adipose-derived mesenchymal stem cells; HGF: hepatocyte growth factor; α-SMA: alpha-smooth muscle actin; Col I: collagen I; Col III: collagen III.
