# Supplementary material for: Paracrine Effects of Adipose-Derived Stem Cells on Matrix Stiffness-Induced Cardiac Myofibroblast Differentiation via Angiotensin II Type 1 Receptor and Smad7
Source: Sci Rep. 2016 Oct 5;6:33067. doi: 10.1038/srep33067 (PMC5050447; doi:10.1038/srep33067)
Supplement: Supplementary Information [file srep33067-s1.pdf]

**Paracrine Effects of Adipose-Derived Stem Cells on Matrix Stiffness-Induced Cardiac Myofibroblast differentiation via Angiotensin II Type 1 Receptor and Smad7**

Kar Wey Yong, Yuhui Li, Fusheng Liu, Bin Gao, Tian Jian Lu, Wan Abu Bakar Wan Abas, Wan Kamarul Zaman Wan Safwani, Belinda Pingguan-Murphy, Feng Xu, Guoyou Huang

## Supplementary Material

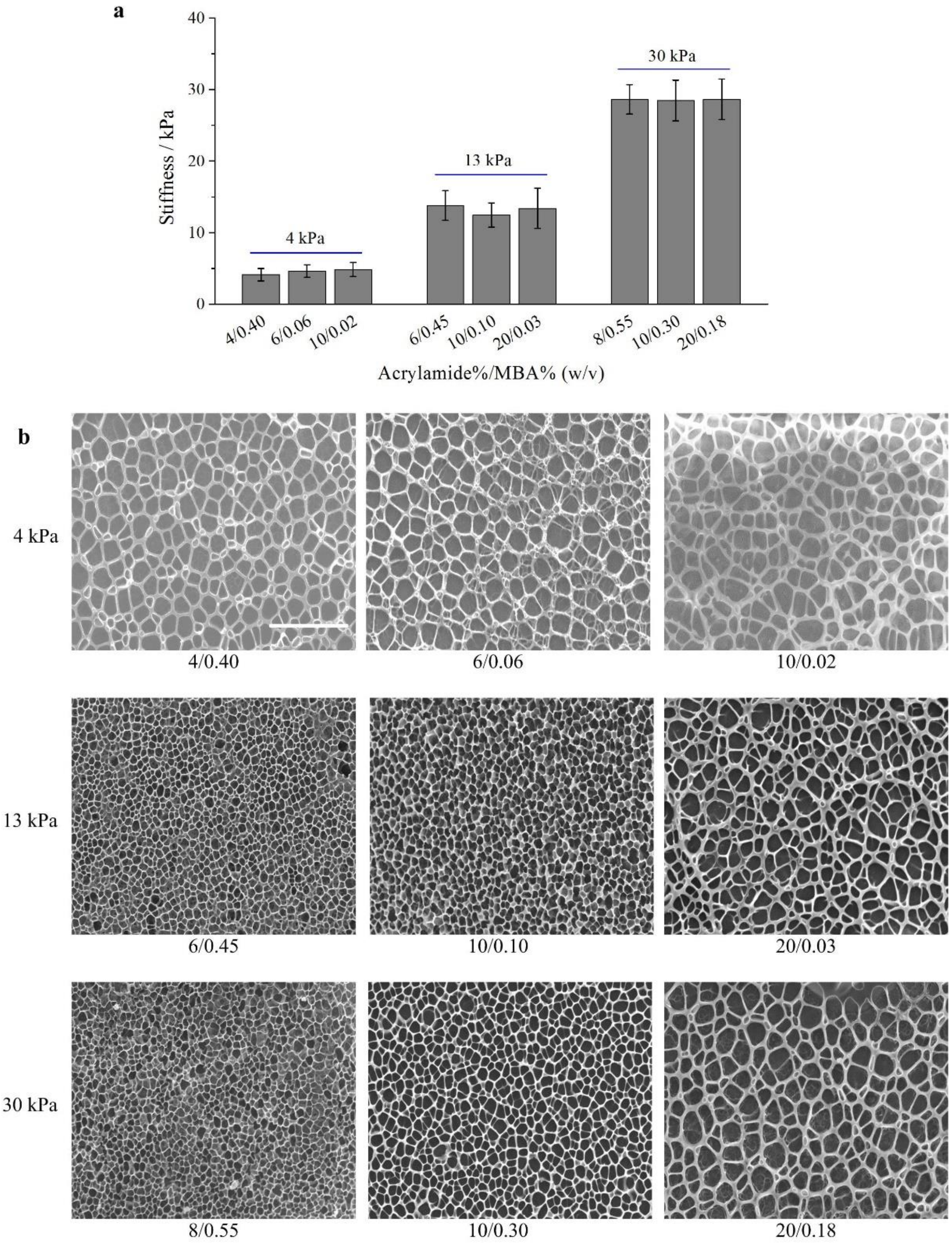


**Supplemental Figure 1. Characterization of PA hydrogels. a)** Stiffness (4 kPa, 13 kPa and 30 kPa) of PA hydrogels made by various ratios of acrylamide:*N*,*N* methylene-bis-acrylamide (MBA) determined by AFM. **b**) Relative pore size observed through SEM increases with increasing concentration of acrylamide and decreasing concentration of MBA for the 4 kPa, 13 kPa and 30 kPa hydrogel formulations. Magnification: 700 ×. Scale bar: 50 μm.

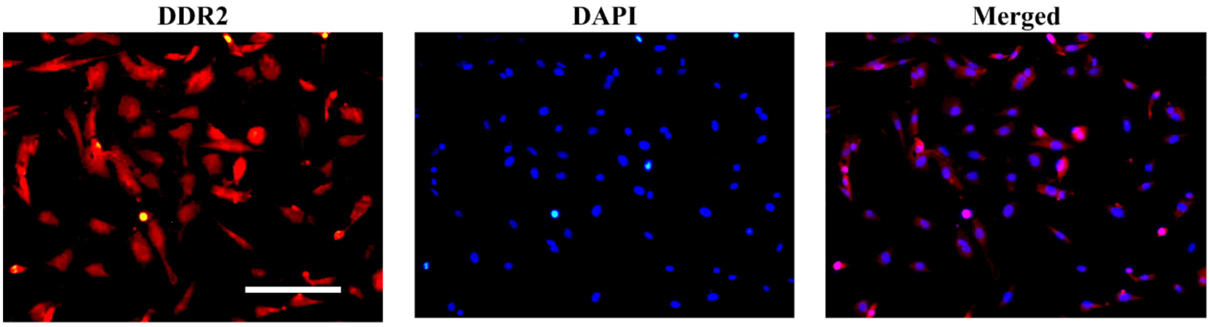


**Supplemental Figure 2**. **Characterization of cardiac fibroblasts**. Almost all the isolated cells displayed DDR2 expression, indicating the presence of only cardiac fibroblasts in the culture. Magnification: 200 ×. Scale bar: 50 µm.

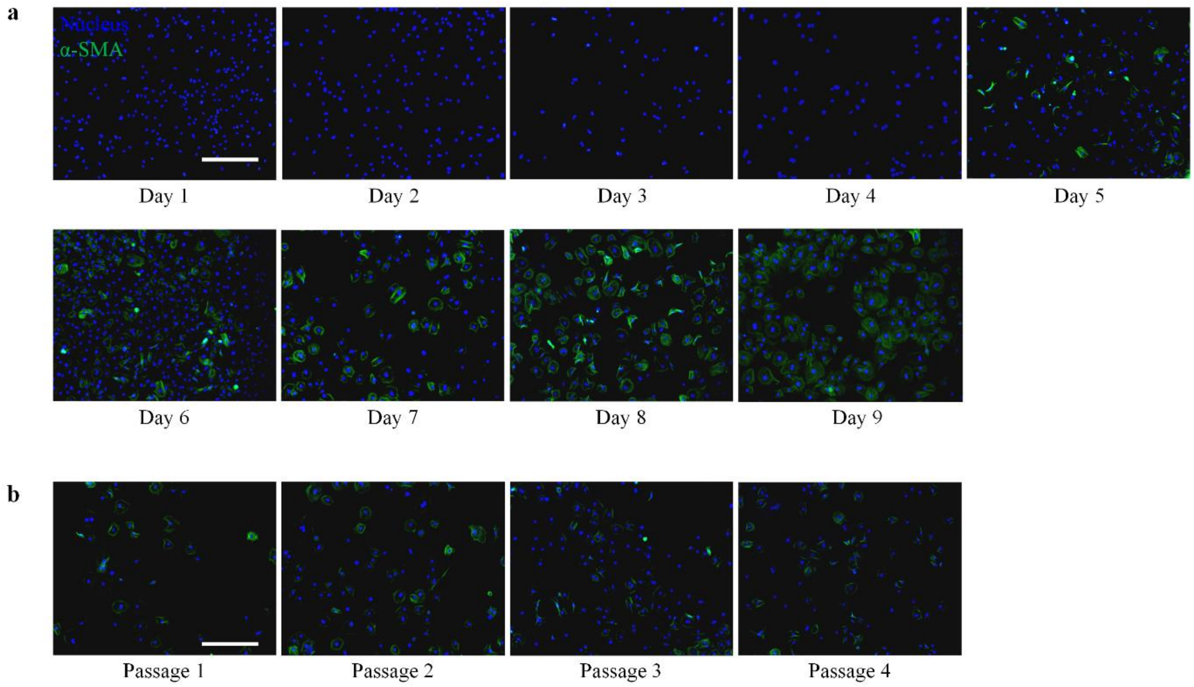


**Supplemental Figure 3. Immunofluorescence staining of cardiac fibroblasts cultured on cell culture plate. a**) Cardiac fibroblasts cultured on cell culture plate before day 5 were negative to alpha-smooth muscle actin (α-SMA), whereas those cultured after day 5 were positively stained by α-SMA. **b**) Cardiac fibroblasts cultured on cell culture plate after day 5 expressed α-SMA regardless of cell passage number. Magnification: 100 ×. Scale bars: 100 μm.

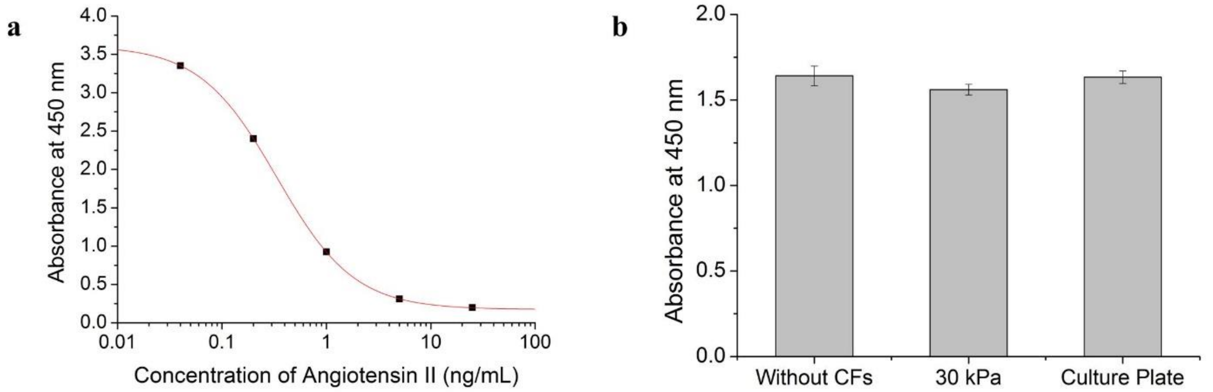


**Supplemental Figure 4. Angiotensin II is not present in the conditioned medium of cardiac fibroblasts (CFs) cultured on 30 kPa substrates and cell culture plate. a)** Standard curve of known concentration of angiotensin II versus absorbance at 450 nm measured through ELISA. **b**) There was no significant difference in the absorbance at 450 nm among the medium without CFs, conditioned medium of CFs cultured on 30 kPa substrates, and conditioned medium of CFs cultured on cell culture plate. After normalization to the control (medium without CFs), it was found that the concentration of angiotensin II in conditioned medium of CFs cultured on 30 kPa substrates and cell culture plate is 0 ng/mL respectively, indicating the absence of angiotensin II.

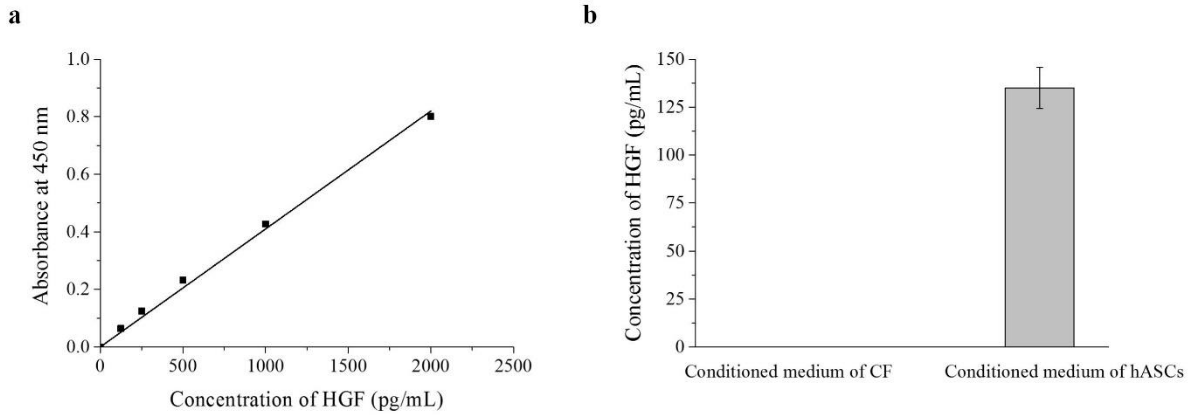


**Supplemental Figure 5**. **Concentration of HGF in conditioned medium of hASCs is 135 ± 10.75 pg/mL. a**) Standard curve of known concentration of HGF versus absorbance at 450 nm measured through enzyme-linked immunosorbent assay (ELISA) with coefficient of determination $R^2$ = 0.99. **b**) Concentration of HGF was significantly higher ($p$<0.05) in conditioned medium of hASCs compared to medium without cells and conditioned medium of cardiac fibroblasts (CFs) (* $p$<0.05 relative to medium without cell; # $p$<0.05 relative to conditioned medium of CFs).
